# Gelatin/hyaluronic acid-based *in situ* forming hydrogel promotes wound regeneration by the synergy of ROS-scavenging and pro-healing activity

**DOI:** 10.1093/rb/rbaf052

**Published:** 2025-06-20

**Authors:** Xingchen Zhao, Wenling Dai, Chenxin Liu, Mei An, Shikui Li, Likun Guo, Yujiang Fan, Xingdong Zhang

**Affiliations:** National Engineering Research Center for Biomaterials, Sichuan University, Chengdu, Sichuan 610064, P.R. China; College of Biomedical Engineering, Sichuan University, Chengdu 610064, P.R. China; National Engineering Research Center for Biomaterials, Sichuan University, Chengdu, Sichuan 610064, P.R. China; College of Biomedical Engineering, Sichuan University, Chengdu 610064, P.R. China; National Engineering Research Center for Biomaterials, Sichuan University, Chengdu, Sichuan 610064, P.R. China; College of Biomedical Engineering, Sichuan University, Chengdu 610064, P.R. China; National Engineering Research Center for Biomaterials, Sichuan University, Chengdu, Sichuan 610064, P.R. China; College of Biomedical Engineering, Sichuan University, Chengdu 610064, P.R. China; National Engineering Research Center for Biomaterials, Sichuan University, Chengdu, Sichuan 610064, P.R. China; College of Biomedical Engineering, Sichuan University, Chengdu 610064, P.R. China; National Engineering Research Center for Biomaterials, Sichuan University, Chengdu, Sichuan 610064, P.R. China; College of Biomedical Engineering, Sichuan University, Chengdu 610064, P.R. China; National Engineering Research Center for Biomaterials, Sichuan University, Chengdu, Sichuan 610064, P.R. China; College of Biomedical Engineering, Sichuan University, Chengdu 610064, P.R. China; National Engineering Research Center for Biomaterials, Sichuan University, Chengdu, Sichuan 610064, P.R. China; College of Biomedical Engineering, Sichuan University, Chengdu 610064, P.R. China

**Keywords:** natural polymer hydrogels, antioxidant activity, wound repair, multinetwork structure, recombinant collagen type III

## Abstract

The development of advanced hydrogel dressings that integrate biocompatibility, antioxidant activity and dynamic adaptability remains critical for addressing the complex demands of modern wound management. In this study, we designed a multinetwork hydrogel (GHrCT) through synergistic strategies: A robust covalent network is constructed through photocrosslinked gelatin methacryloyl, while a secondary dynamic network formed via hydrogen bonds and electrostatic interactions is established among dopamine-modified hyaluronic acid (HD), tannic acid (TA) and recombinant collagen type III (rhCol III). Through a series of experiments, we systematically characterized key properties of the hydrogel, including its microscopic morphology, swelling behavior, rheological characteristics and mechanical strength. Biocompatibility was assessed through *in vitro* assays, while the wound healing efficacy was validated *in vivo*. *In vitro* experiments demonstrated that GHrCT hydrogel has interconnected porosity, excellent hemocompatibility and good cytocompatibility. Its strong antioxidant capacity (DPPH scavenging rate of 88.63%) can cope with the excessive accumulation of ROS in the wound microenvironment and reduce the damage caused by oxidative stress. Further, *in vivo* experiments showed that it could improve wound healing therapy by accelerating epithelial re-formation, angiogenesis and collagen deposition at full-thickness skin defects in SD rats. This study presents a strategy for functionalizing natural polymer hydrogels to enhance wound repair through the synergistic effect of scavenging ROS and promoting repair.

## Graphical Abstract

**Figure:**
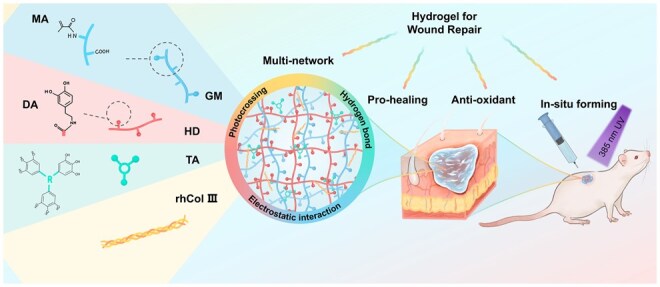

## Introduction

The skin, as the largest organ of the human body, serves as the primary protective barrier between the internal and external environments. This multifunctional organ provides essential physiological protection through its dual defense mechanisms: not only does it defend against external threats including mechanical trauma, chemical exposure and microbial invasion but it also maintains internal homeostasis by preventing excessive transepidermal water loss [1]. Consequently, any impairment to the skin’s structural integrity or physiological functions can significantly compromise overall health and increase susceptibility to various medical complications [2]. Appropriate external treatments can significantly enhance and accelerate the healing process [3–5].

Conventional wound dressings, such as gauze and bandages, often fail to address the dynamic requirements of the wound healing microenvironment, particularly in balancing mechanical properties, oxidative stress mitigation and bioactive support [6–8]. In contrast, hydrogel dressings exhibit distinct advantages due to their excellent biocompatibility and three-dimensional porous architecture mimicking the extracellular matrix (ECM). Hydrogels not only physically protect wounds by providing an antimicrobial barrier but also function as bioactive scaffolds to guide the migration and reorganization of dermal cells. Furthermore, their ECM-like structure facilitates nutrient transport and cellular interactions, demonstrating significant potential in promoting coordinated wound healing processes [7].

Natural polymers are macromolecular compounds derived from biological sources (plants, animals, microorganisms) through extraction or biosynthesis, with tunable physicochemical properties achievable via controlled processing techniques [9]. Among these, natural polymer-based hydrogels (e.g. cellulose, hyaluronic acid, sodium alginate and collagen) exhibit inherent advantages including renewability, biodegradability and biocompatibility, positioning them as pivotal biomaterial candidates for advanced wound healing applications [10–12]. Hyaluronic acid (HA), a glycosaminoglycan ubiquitously present in connective tissues, plays critical physiological roles in tissue hydration, nutrient transport, proteoglycan assembly and cellular differentiation [13]. Despite these biological merits, HA-based hydrogels exhibit two critical limitations: compromised mechanical integrity under high hydration due to excessive water absorption, combined with insufficient cell adhesion capacity stemming from the absence of specific ligand-binding domains in their molecular architecture. These inherent constraints collectively hinder cellular adhesion-migration dynamics essential for tissue regeneration [14, 15]. Gelatin (Gel), a hydrolytic derivative of collagen, demonstrates excellent biocompatibility and biodegradability due to its structural similarity to natural extracellular matrix components [16, 17]. This biomaterial exhibits low immunogenicity while retaining bioactive motifs analogous to the arginine-glycine-aspartate (RGD) sequence, which facilitates cellular adhesion, migration, differentiation and proliferation through integrin-mediated signaling pathways [18, 19]. The presence of abundant amino groups on its side chains enables functional modification via methacrylation, yielding methacryloyl gelatin (GM) with photosensitive properties [20]. Hydrogels fabricated via this method enable *in situ* photocrosslinking, a feature that has driven their widespread adoption in tissue engineering and wound repair applications. Previous studies have demonstrated that the synergistic combination of HA and gelatin can amplify their individual advantages while mitigating inherent limitations through the incorporation of bioactive components. This integrative approach not only enhances hydrogel functionality but also enables tailored therapeutic effects for wound repair. Numerous functionalized hydrogels based on gelatin/hyaluronic acid (Gel/HA) systems have been developed to enhance wound healing [21–24].

It should be noted that there is a significant amount of reactive oxygen species (ROS) production in injured tissues [25]. However, the imbalance between ROS generation and antioxidant clearance capacity often results in excessive ROS accumulation, a pathological condition termed oxidative stress. This oxidative imbalance exerts multiple detrimental effects on the wound healing process, including cellular damage and impaired tissue regeneration [26–28]. Therefore, when engineering hydrogel-based systems for cutaneous wound repair, particular emphasis must be placed on mitigating oxidative stress-induced damage to ensure optimal therapeutic outcomes. The inherent antioxidant properties of natural polyphenols have emerged as a potent strategy to enhance ROS scavenging and mitigate oxidative stress-related tissue damage, garnering significant research interest in recent years. Functional hydrogels rich in catechol moieties have been shown to promote wound healing by eliminating ROS and modulating the wound microenvironment [29, 30]. Tannic acid (TA), a hydrolysable gallotannin composed of multiple galloyl glucose units, provides abundant hydrogen bond donors/acceptors that enable polyphenol-protein complexation. This molecular architecture not only allows efficient ROS neutralization for antioxidant and anti-inflammatory effects but also promotes endothelial cell proliferation and angiogenesis [31, 32]. In parallel, dopamine (DA)—a catecholamine containing both catechol and amino functional groups—exhibits surface-rich reactive moieties that facilitate adsorption of ECM components (e.g. fibronectin), thereby accelerating wound re-epithelialization through enhanced cell-matrix interactions [33, 34]. The dopamine-modified hyaluronic acid (HD) maintains a moist wound environment while forming stable adhesion interfaces to promote cell spreading and migration [35]. Its hydrogen bond-mediated energy dissipation mechanism enhances mechanical stability and shape adaptability. Moreover, unlike physical mixtures, the intramolecular hydrogen bonds enable long-term oxidative stress resistance [36, 37]. Type III collagen (Col III), the predominant collagen subtype in early-stage granulation tissue, plays a critical role in accelerating wound closure through its unique fibrillar organization that facilitates provisional matrix formation [38]. Recombinant collagen (rhCol)—engineered through codon-optimized gene sequences that preserve the functional domains of human collagen while enhancing stability—exhibits distinct advantages over animal-derived collagens. These include batch-to-batch consistency, reduced immunogenicity and improved aqueous solubility, addressing key limitations of traditional collagen biomaterials [39, 40]. Zhang *et al* further demonstrated that recombinant collagen type III (rhCol III) containing specific amino acids (serine, threonine and tyrosine) can scavenge ROS through redox reactions, thereby effectively reducing oxidative stress while protecting collagen from ROS-induced degradation [41]. Recent studies have shown that rhCol III promotes the synthesis of native Col III through endocytosis by fibroblasts and promotes wound healing and reduces scar proliferation [42]. This dual-action mechanism enhances the wound healing process.

In summary, developing natural polymer-based hydrogels that integrate exceptional biocompatibility with robust antioxidant activity represents a critical yet underexplored strategy for addressing the complex demands of modern wound healing. In this study, we designed a multinetwork antioxidant hydrogel with *in situ* forming capability, designated as GHrCT. The hydrogel fabrication involved two principal strategies: (1) The photocrosslinked GM hydrogel forms an irreversible primary network to achieve complete filling of irregular wounds; (2) The incorporation of hydroxyl-rich tannic acid (TA) and hydrogen bonding between phenolic hydroxyl groups of HD and the carboxyl/amino groups of GM and rhCol III collaboratively establish a secondary noncovalent network, achieving synergistic structural and functional integration. The GHrCT hydrogel exhibited an interconnected porous architecture, optimal swelling behavior, tunable mechanical properties and exceptional biocompatibility. Notably, its potent ROS-scavenging capacity synergistically promoted re-epithelialization and collagen deposition, ultimately accelerating coordinated wound repair through oxidative stress mitigation and cellular microenvironment regulation.

## Materials and methods

### Materials

Gelatin (Gel) from pig skin source was purchased from Sigma-Aldrich Corporation (USA). Dopamine hydrochloride (DA) and tannic acid (TA) were sourced from Shanghai Macklin Biochemical Technology Co., Ltd (China). Sodium hyaluronate (HA, Mw 1400 kDa) was provided by Bloomage Biotechnology Co., Ltd. (China). The crosslinking agents 1-ethyl-3-(3-dimethylaminopropyl) carbodiimide hydrochloride (EDC) and N-hydroxysuccinimide (NHS) as well as methacrylic anhydride (MA) were obtained from Shanghai Titan Scientific Co., Ltd. (China). Recombinant collagen type III (rhCol III) was supplied by Jiangsu Trautec Medical Technology Co., Ltd. (China).

### Synthesis of HD

HA was dissolved in deionized water to form a 1% (w/v) aqueous solution. NHS and EDC were added to the solution and the pH was adjusted to 5.0. Allow the reaction to proceed in the dark for 30 min. Then, DA was added to the solution and the reaction continued in the dark for 24 h, with argon gas protection throughout. The reaction product was dialyzed in acidic solution for 3 days, and then, dialyzed in water for 4 days, using a dialysis bag with a molecular weight cut-off of 8000–14 000 Da. Finally, the purified product was freeze-dried and named HD.

### Synthesis of GM

Gelatin (10 g) was dissolved in a Na_2_CO_3_-NaHCO_3_ buffer solution to form a 10% (w/v) aqueous solution. The pH of the solution was adjusted to 9.0 at 50°C. MA (1 mL) was added dropwise to the solution and the reaction was allowed to proceed for 4 h. The product was precipitated using anhydrous ethanol at 4°C. The mixture was centrifuged at 3000 rpm for 15 min and the supernatant was discarded. The precipitate was redissolved in deionized water. The solution was dialyzed in the dark at 37°C for 5 days, using a dialysis bag with a molecular weight cut-off of 8000–14 000 Da. Finally, the purified product was freeze-dried and named GM.

### Characterization of HD and GM

The structural characterization of HD was performed using Fourier Transform Infrared (FTIR) spectroscopy. The formation of HD was confirmed by ultraviolet-visible (UV–Vis) spectroscopy (240–400 nm), while structural modifications of HD and GM were characterized via proton nuclear magnetic resonance hydrogen spectrum (^1^H NMR) spectroscopy in D_2_O.

### Fabrication of GHrCT hydrogels

Briefly, HD, GM, rhCol III and TA were dissolved in PBS buffer solution, respectively. The resulting solutions were then mixed uniformly using magnetic stirring to prepare the hydrogel precursor solution according to the concentration ratios shown in Table 1. The pH of mixed solution was adjusted to neutral using 1M NaOH solution and 1M HCl solution. After exposed to 385 nm light for 1 min, the hydrogels formed *in situ* and were designated as GH, GHrC and GHrCT, respectively. Based on different concentrations of TA, the GHrCT hydrogels were distinguished as GHrCT1 and GHrCT2.

**Table 1.** Composition of hydrogels

	GM	HD	rhCol III	TA
GH	10% (w/v)	1% (w/v)	0	0
GHrC	10% (w/v)	1% (w/v)	0.4% (w/v)	0
GHrCT1	10% (w/v)	1% (w/v)	0.4% (w/v)	0.1% (w/v)
GHrCT2	10% (w/v)	1% (w/v)	0.4% (w/v)	0.2% (w/v)
GHrCT4	10% (w/v)	1% (w/v)	0.4% (w/v)	0.4% (w/v)
GHrCT8	10% (w/v)	1% (w/v)	0.4% (w/v)	0.8% (w/v)

### Micromorphology of hydrogels

The microstructure of hydrogels was observed using scanning electron microscopy (SEM). The hydrogels were rapidly frozen in liquid nitrogen for structural preservation, followed by freeze-drying for 48 h. Cross-sectional surfaces were obtained through liquid nitrogen-assisted fracture. Subsequently, the dried hydrogel samples were sputter-coated with gold. Finally, the morphology of the freeze-dried hydrogel samples was examined using a SEM.

### Swelling rate testing

The hydrogel samples were placed in PBS solution and incubated at 37°C. At the predetermined time intervals, the hydrogels were removed from the solution and the surface moisture was blotted off using filter paper. The hydrogels were then weighed. The test was completed when the weight of all hydrogels remained constant. Each set of measurements was performed in triplicate.

The calculation method for the swelling ratio was as follows:


$$\text{Swelling}\;\text{Ratio}\;=\;(\frac{W_{t}-W_{0}}{W_{0}})\times 100\%.$$

Here, *W*_0_ and *W_t_* represent the initial weight of the hydrogel and the weight after the preset swelling time, respectively.

### Mechanical properties of hydrogels

The compressive stress–strain behavior was characterized using a dynamic mechanical analyzer (DMA) under ambient temperature conditions. All hydrogel samples were prepared in a cylindrical shape (∼2.0 mm in height × 8.0 mm in diameter). The stress was measured within a range of 1 mN–4 N at a rate of 0.5 N/min. The elastic modulus was determined from the slope of the linear elastic region (0–10% strain) in the stress–strain curve.

### Rheological properties of hydrogels

The tests were conducted using a rotational rheometer at room temperature. The hydrogel samples were prepared in a cylindrical shape (∼2 mm in height × 25.0 mm in diameter). Strain sweeps were performed at a constant frequency of 1 Hz over a strain range of 0.1–100%. Frequency sweeps were conducted at a constant strain of 1% over a frequency range of 0.1–100 Hz. At least three parallel samples were used for each group.

### Degradation properties of hydrogels

The *in vitro* degradation behavior of hydrogels was investigated through co-incubation with 0.25% (w/v) trypsin at 37°C. The initial dry mass of hydrogels was recorded as *w*_0_, while the residual dry mass after incubation for 0, 30, 60, 90, 120, 150, 180 and 240 min was denoted as *w_t_*. The percentage of mass remaining was used to reflect the degradation properties of the hydrogel:


$$\text{Mass}\;\text{remaining}\;=\;\frac{w_{t}}{w_{0}}\times 100\%.$$

### Release of rhCol III and TA from hydrogels

The cumulative release profiles of rhCol III and TA from hydrogels were quantitatively analyzed using UV–Vis spectrophotometry. Standard concentration-absorbance curves for both components were first established through linear regression analysis. Hydrogel samples from each group were immersed in 1 mL of PBS (pH 7.4) at 37°C. At predetermined intervals (1, 2, 3, 4, 24, 48, 72, 96, 120, 144, 168, 192, 216 and 240 h), the supernatant was collected and replaced with an equal volume of fresh PBS. The released concentrations of rhCol III and TA at each time point were determined by correlating the measured absorbance values with their respective standard curves.

### Cytocompatibility evaluation of hydrogels

Cell compatibility experiments were conducted using mouse fibroblast cell line L-929 (L929) for CCK-8 assays and live/dead cell detection. L929 cells were seeded at a density of 0.1 mL (5 × 10^3^ cells/mL) into a 96-well plate and incubated overnight. Sterile hydrogel samples were prepared and co-cultured with DMEM medium at a ratio of 0.1 g hydrogels/mL for 24 h to obtain the extract. The complete medium was then replaced with an equal volume of the extract containing 10% serum and further cultured. Cell proliferation was assessed on Days 1, 2 and 3 using the CCK-8 method by measuring absorbance at 450 nm. Cell viability was evaluated on Days 1, 3 and 5 using fluorescein diacetate and propidium iodide (FDA/PI) staining, and observed under an inverted fluorescence microscope.

### Cell scratch assay

L929 cells were seeded in 6-well plates at a density of 5 × 10^5^ cells/well with 2 mL culture medium, followed by incubation at 37°C in a 5% CO_2_ humidified atmosphere. When the cells reached 90% confluence, a uniform scratch was made using a sterile pipette tip. The cells were then washed three times with PBS to remove detached cells. The complete medium was replaced with an equal volume of different extracts containing 10% serum and further cultured. Cell migration was observed and photographed at 0, 6, 12 and 24 h using a inverted fluorescence microscope to record the migration of cells in each group.

### Hemocompatibility of hydrogels

4 mL of anticoagulated blood were added to a centrifuge tube containing 5 mL of physiological saline and the mixture was gently mixed to prepare diluted blood for the hemolysis experiment. Physiological saline served as the negative control, while deionized water was used as the positive control. One hundred milligrams of hydrogel were added to a centrifuge tube containing 1 mL of physiological saline and the mixture was incubated at 37°C for 1 h. Twenty microliters of the diluted blood were then added to the centrifuge tube and the mixture was further incubated at 37°C for another hour. The hydrogel was carefully removed and the sample was immediately centrifuged at 1500 rpm for 5 min, followed by the capture of an optical photograph. One hundred microliters of the supernatant were transferred to a 96-well plate, and the absorbance of hemoglobin at 545 nm was measured using a microplate reader. The hemolysis rate was calculated using the following formula:


$$\text{Hemolysis}\;\text{rate}=\frac{\left({\text{OD}}_{\text{sample}}-{\text{OD}}_{\text{negative}}\right)}{\left({\text{OD}}_{\text{positive}}-{\text{OD}}_{\text{negative}}\right)}\times 100\%.$$

Here, OD_sample_, OD_negative_ and OD_positive_ represent the absorbance values of the experimental group, negative control group and positive control group, respectively.

### Antioxidant properties of hydrogels

#### Intracellular ROS scavenging assay

The ability of the hydrogel to scavenge intracellular ROS was investigated using the ROS probe DCFH-DA. L929 cells were seeded at a density of 5 × 10^4^ cells per well in a 24-well plate and incubated overnight in a cell culture incubator. The DCFH-DA probe was loaded, and the cells were incubated in the dark in the cell culture incubator for 40 min. The supernatant was discarded, and the cells were washed three times with PBS to remove any unloaded DCFH-DA. The negative control group was cultured with complete medium, the positive control group was cultured with complete medium containing 100 μM H_2_O_2_, and the experimental group was cultured with hydrogel extract containing 100 μM H_2_O_2_ and 10% serum. After 12 h of incubation, the supernatant was discarded and the cells were washed with PBS. The cells were then observed under an inverted fluorescence microscope, and the fluorescence intensity was captured and quantitatively analyzed using ImageJ.

#### DPPH assay

An ethanolic DPPH• radical solution (100 μM) was freshly prepared according to Blois method [43]. Hydrogel samples (0.1 g) were immersed in 1 mL DPPH• solution before incubation at 37°C under light-protected conditions for 45 min. After centrifugation, 100 μl supernatant was aliquoted into 96-well microplates. Absorbance measurements at 517 nm were measured using a microplate reader. Radical scavenging activity (%) was calculated using:


$$\text{DPPH}\;\text{scavenging}=(1-\frac{A_{\text{sample}}}{A_{\text{control}}})\times 100\%,$$

where *A*_control_ represents absorbance of DPPH• solution without sample.

#### Scratch assay under oxidative stress

L929 cells were plated in 6-well culture dishes at 5 × 10^5^ cells/well (2 mL medium/well) and maintained at 37°C in a 5% CO_2_ atmosphere. When the cells reached 90% confluence, a uniform scratch was made using a sterile pipette tip. The cells were then washed three times with PBS to remove detached cells. The positive control group was cultured in complete medium containing 100 μM H_2_O_2_, while the experimental group was cultured in hydrogel extracts containing 100 μM H_2_O_2_ and 10% serum. For the experimental group, the complete medium was replaced with different extracts of equal volume containing 10% serum and 100 μM H_2_O_2_. Cell migration was observed and photographed using a inverted fluorescence microscope at 0, 12, 24 and 48 h, and the migration of cells in each group was recorded.

### *In vivo* wounding healing assessment

The animal study was approved by the Experimental Animals Ethics Committee of Sichuan University (No. K2023014). The animal experiment guidance from the ethical committee and the guide for care and use of laboratory animals of Sichuan University were followed during the whole experiment course. A full-thickness skin defect model was constructed using male SD rats (weighing 220–250 g) to assess the effect of hydrogels on *in vivo* wound healing. All animal use, care and procedures were conducted at the Animal Experiment Center of Sichuan University. Following dorsal depilation, a full-thickness circular skin defect (10 mm diameter) was surgically created on rat dorsum. A silicone ring (10 mm inner diameter) was circumferentially secured to the wound periphery to inhibit contraction. The hydrogel precursor solution was injected into the wound bed and photo-crosslinked via 385 nm blue light irradiation to achieve *in situ* polymerization. A sterile gauze barrier was applied to prevent mechanical disruption of the hydrogel dressing. Control wounds received sterile gauze coverage only. Wound photographs and tissue samples were collected on Days 0, 3, 7 and 14. Samples of major organs (heart, kidney, liver, lung and spleen) were collected from rats on Day 21. Tissues were fixed in 4% paraformaldehyde solution. Subsequently, the wound tissues were embedded in paraffin, sectioned and further analyzed. The sections were stained using hematoxylin-eosin (H&E) staining kit, Masson’s trichrome staining kits and Sirius Red staining kits.

### Statistical analysis

*T*-tests, one-way ANOVA and two-way ANOVA were performed using GraphPad Prism 8 software to compare paired samples and multiple groups. Generally, significance levels were defined as follows: *P <* *0.05, **P <* *0.01 and ***P <* *0.001. All experimental samples were analyzed in triplicate or more.

## Results and discussion

### Preparation and characterization of HD, GM and GHrCT hydrogel

A series of wound-healing hydrogels with antioxidant properties were synthesized and systematically characterized. The physical properties of hydrogels (micromorphological features, swelling behavior and rheological characteristics) were evaluated. Furthermore, their biocompatibility and antioxidant activities were thoroughly investigated to assess their potential applications in skin wound healing.

**Figure 1.**
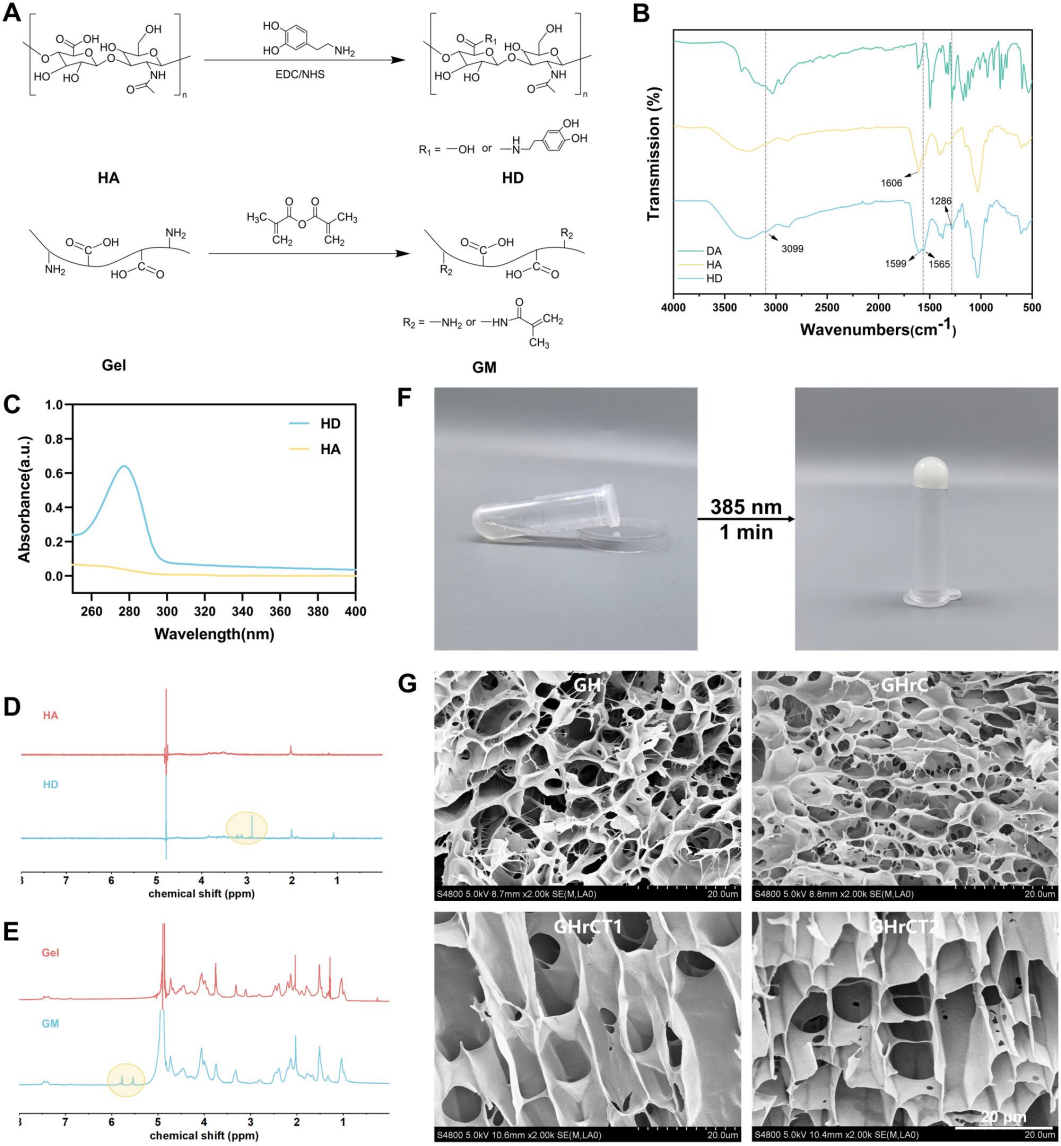
Material synthesis and hydrogels preparation. (**A**) Synthesis diagram of HD and GM. (**B**) FTIR spectra of DA, HA and HD. (**C**) UV–Vis spectra of HA and HD. (**D**) ^1^H NMR spectra of HA and HD. (**E**) ^1^H NMR spectra of Gel and GM. (**F**) Formation process of GHrCT. (**G**) Microscopic morphology of hydrogels.

HD and GM were synthesized as shown in Figure 1A, and characterized using Fourier transform infrared spectroscopy, ultraviolet–visible spectroscopy and proton nuclear magnetic resonance spectroscopy. In the FTIR spectra shown in Figure 1B, the asymmetric stretching vibration peak of the carboxylate group (-COO^−^) in HD red-shifted from 1606 cm^−1^ (HA) to 1599 cm^−1^ with peak broadening, suggesting the involvement of carboxylate groups in the reaction and the formation of a new chemical environment. Characteristic peaks corresponding to the amide II band (N-H bending and C-N stretching vibrations) and amide III band (C-N stretching and N-H bending vibrations) were observed in the FTIR spectra of HD at 1565 cm^−1^ and 1286 cm^−1^, respectively. Additionally, a newly emerged aromatic C-H stretching vibration peak at 3099 cm^−1^ indicated the integration of dopamine’s catechol structure into the HA backbone. These spectral changes collectively demonstrated the successful covalent grafting of DA onto HA through amide bond formation. In the UV–Vis spectra of HA and HD as shown in Figure 1C, a new absorption peak at 280 nm was observed, indicating the successful graft of DA onto HA [32, 44]. Based on the 280 nm wavelength of HD, the degree of substitution (DS) of DA in HD was calculated to be approximately 15.3%. In addition, the absence of an absorption peak at 395 nm indicated that the catechol moiety in HD was not oxidized to quinone [45]. The ^1^H NMR spectrum of HD as illustrated in Figure 1D exhibited new chemical shifts at 2.93 ppm which was assigned to the -CH_2_- group adjacent to the catechol ring. These findings further confirmed the grafting of DA onto HA. MA underwent an amidation reaction with the amino groups present in the Gel molecular chain, resulting in the formation of GM. Two distinct absorption peaks appeared at 5.43 ppm and 5.67 ppm in the ^1^H NMR spectrum of Gel and GM (Figure 1E). These characteristic peaks corresponded to the vinyl protons (-C=CH_2_) of the methacryloyl group introduced from MA. The DS of MA in GelMA was calculated to be 88.1% by ^1^H NMR.

The hydrogel precursor demonstrated the capability to form strong and irreversible chemical bonds through photocrosslinking upon brief irradiation with 385 nm blue light (Figure 1F). Concurrently, the incorporation of polyphenols facilitated the formation of dynamic hydrogen bonds within hydrogel matrix, thereby establishing a dynamic secondary network. Furthermore, the *in situ* forming characteristics of GM enabled the hydrogel to precisely conform to irregular tissue surface geometries. The microscopic morphology of the hydrogels was characterized using SEM and the results were shown in Figure 1G, which revealed that all hydrogels exhibited a homogeneous porous structure. This structural characteristic is essential to facilitate the transport of nutrients and oxygen, thereby supporting cell adhesion, migration and normal cellular functions [46].

### Characteristics of hydrogels

**Figure 2.**
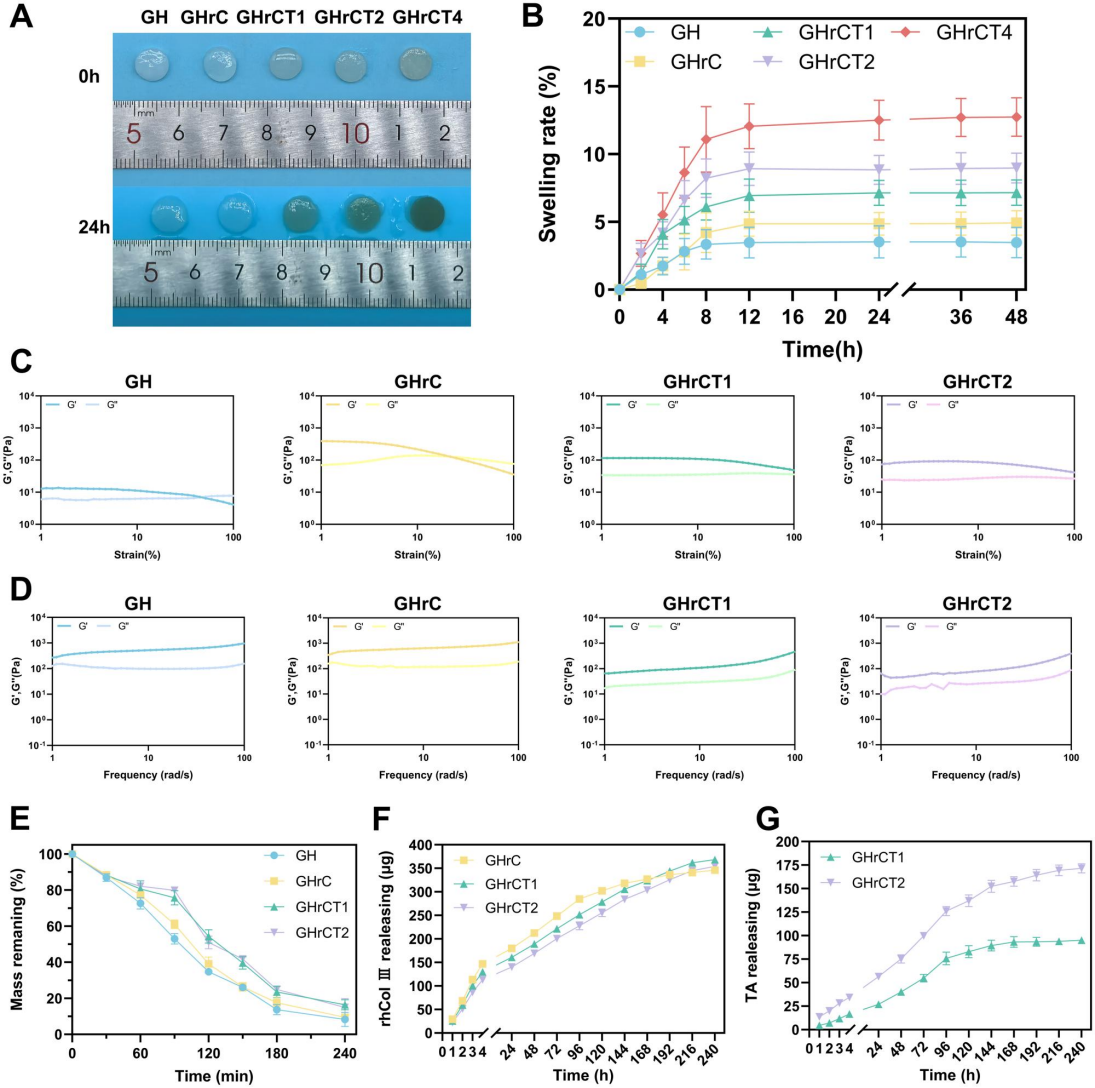
Characterization of physicochemical properties of hydrogels. (**A**) Swelling behavior of hydrogels. (**B**) Swelling rate of hydrogels. Rheological properties of hydrogels, including (**C**) frequency scans and (**D**) strain scans. (**E**) Proteolytic degradability of hydrogels. (**F**) Release behavior in hydrogels of rhCol III. (**G**) Release behavior in hydrogels of TA.

The swelling behavior of hydrogels is governed by osmotic pressure gradients between their polymer networks and surrounding physiological environments, such as those encountered in cutaneous wound beds [47, 48]. These environments are characterized by the presence of water-rich media with low osmotic pressure. Prolonged exposure to such wet conditions can compromise hydrogel network, leading to structural instability [49]. Hydrogels with optimized swelling properties demonstrate the capacity to absorb wound exudate while maintaining an optimal moist environment that facilitates wound healing. Figure 2A and B presented a comparative analysis of swelling characteristics and quantitative swelling ratios across hydrogel formulations. All hydrogel formulations reached swelling equilibrium within 24 h at physiological temperature (37°C). Notably, the GH hydrogel demonstrated the lowest swelling capacity (equilibrium swelling ratio of 7.14%), attributed to its highly crosslinked rigid network that restricted water infiltration. The incorporation of hydrophilic rhCol III in GHrC hydrogels significantly enhanced hydration capacity through enhanced water-binding interactions. In contrast, the GHrCT hydrogels showed concentration-dependent swelling characteristics, with swelling rates ranging from 7.14% to 12.51% that correlated with TA concentration gradients. This phenomenon stems from the energy dissipation mechanism created by the formation of a dynamic hydrogen bond network [50]. The swelling behavior dictated by the multinetwork architecture of GHrCT hydrogel exhibited tripartite functionality: Not only does it sustain a moisture-rich microenvironment optimal for wound healing, but it also reduces secondary injury risks through interfacial friction suppression and simultaneously reinforces the hydrogel’s structural integrity.

The rheological properties of GH, GHrC, GHrCT1 and GHrCT2 hydrogels were systematically investigated through the measurement of storage modulus (G′') and loss modulus (G″) under varying frequency and strain conditions. As shown in Figure 2C, the frequency sweep analysis revealed that GH and GHrC hydrogels exhibited similar rheological behavior patterns. The GHrCT1 hydrogel demonstrated slightly reduced G′ and G″ values while maintaining parallel curves throughout the frequency range, indicating that this formulation could provide necessary mechanical support while exhibiting improved flexibility and tissue compliance. In contrast, the GHrCT2 hydrogel showed fluctuations in the G″ curve, which could be attributed to the higher TA content facilitating the formation of additional dynamic hydrogen bonds, potentially leading to localized network instability. Strain scan analysis further demonstrated distinct mechanical characteristics among the hydrogel groups. The GH hydrogel exhibited the lowest G′ and G″ values, suggesting good extensibility but potentially insufficient mechanical strength, likely resulting from its relatively simple network structure. The GHrC hydrogel displayed higher modulus values and a lower crossover strain point, indicating a denser network structure with enhanced mechanical strength but reduced deformation resistance. Both GHrCT1 and GHrCT2 hydrogels showed similar rheological characteristics in strain sweep tests (Figure 2D), demonstrating excellent mechanical stability and structural homogeneity. Comprehensive rheological analysis revealed that the GHrCT1 hydrogel, with its optimized multiple network structure, achieved the best balance between mechanical properties and structural stability. This balanced performance, combined with appropriate tissue compliance, makes the GHrCT1 formulation particularly suitable for wound healing applications.

*In vitro* enzymatic degradation of various hydrogels were systematically investigated, as illustrated in Figure 2E. GHrCT1 and GHrCT2 hydrogels exhibited similar biphasic degradation patterns characterized by an initial slow phase followed by accelerated degradation, distinct from the monotonic degradation observed in GH and GHrC hydrogels. This phenomenon could be attributed to the hydrogen bond-rich networks in GHrCT hydrogels that initially dominated the swelling behavior during the early degradation stage, thereby delaying enzymatic penetration and resulting in reduced degradation rates [51, 52]. However, as progressive disruption of hydrogen bonds occurred, the structural integrity became compromised, allowing enhanced enzyme accessibility to susceptible chemical bonds within the hydrogel matrix and consequently triggering rapid degradation. The superior degradation performance of GHrCT hydrogels originates from their rationally designed multinetwork architecture, which synergistically enhances structural stability while achieving optimized degradation kinetics through sequential network dissociation mechanisms.

**Figure 3.**
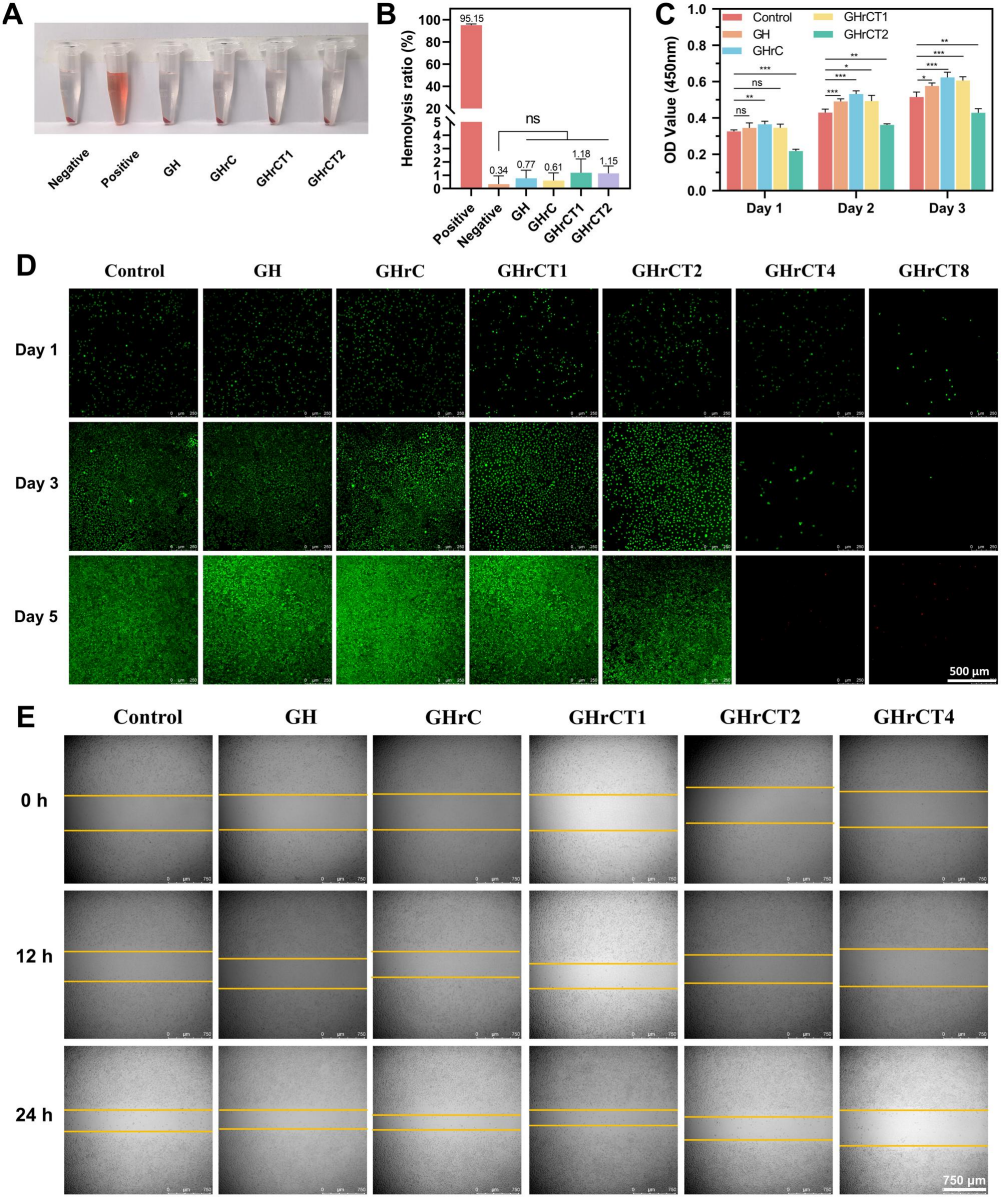
Biocompatibility of hydrogels. (**A**) Hemolysis photographs of hydrogels. (**B**) Hemolysis rate of hydrogels. (**C**) CCK-8 viability assay of L929 cells cultured with hydrogel aqueous extracts. (**D**) Live/dead staining of L929 cells. (**E**) Cell migration of L929 cells.

Small molecules in the hydrogel, including rhCol III and TA, are released into the wound site to provide antioxidant protection and promote wound healing. Figure 3F and G illustrated the release profiles of rhCol III and TA from different hydrogels. The differential release kinetics of rhCol III and TA from hydrogels arise from architecturally regulated molecular interactions, where GHrCT's multinetwork structure enables spatiotemporal control through dynamic hydrogen bonding and hydrophobic effects. Initial burst release of rhCol III (0–4 h) across all groups originated from surface molecule diffusion and swelling-induced porosity. Subsequently, the relatively single covalent network of the GHrC group lacked hydrogen bond-mediated interactions and exhibits rhCol III rapid release (4–96 h) and subsequent slowing of the release (>120 h) of rhCol III [53]. In contrast, GHrCT hydrogels sustained rhCol III delivery via hydrogen bond reorganization that stabilizes porous frameworks. Relatedly, TA demonstrated biphasic release properties in GHrCT hydrogels: rapid release (0–96 h) from weakly bound superficial domains transitioned to retarded diffusion (>96 h) as deeper TA molecules overcame the dense hydrogen-bonded networks. Notably, the regulated release of GHrCT hydrogels is consistent with the requirements of wound healing, i.e., the early and rapid release of TA to counteract the rapid accumulation of ROS in the early stages of wound formation, while the uniform and sustained release of rhCol III continues to play a role in all stages of wound healing [54].

### Biocompatibility of hydrogels

For hydrogels applied in wound repair, good hemocompatibility can mitigate inflammatory responses, reduce the risk of infection and maintain the stability of wound microenvironment, thereby promoting vascular regeneration during the healing process [55, 56]. Hemolysis experiments were conducted to evaluate the hemocompatibility of the hydrogels. As shown in Figure 3A, the positive control group (deionized water) exhibited significant hemolytic activity, as indicated by the bright red color. In contrast, the negative control group (physiological saline) and all hydrogel experimental groups demonstrated minimal hemolytic rate. The hemolysis rate of all tested hydrogels remained below the 5% ISO biocompatibility threshold (Figure 3B), indicating that hemocompatibility met the clinical requirements for hematology applications [57, 58].

For biomedical materials, good biocompatibility is essential to prevent adverse reactions and support long-term functionality in biological environments. Fibroblasts, as a key cell type in wound repair and regeneration, play a crucial role in the skin healing process through regulation of ECM secretion. The cytotoxicity of the hydrogels on L929 was evaluated using the CCK-8 assay and FDA/PI staining. As shown in Figure 3C, the proliferation of L929 cells was assessed on Days 1, 2 and 3. Compared with the control group, the GHrC group demonstrated enhanced cell proliferation on Day 1 (*P *<* *0.01), while GHrCT2 exhibited significant inhibition (*P *<* *0.001). On Day 2, both GH and GHrC groups showed markedly increased proliferation (*P *<* *0.001), with GHrCT1 beginning to display promotive effects (*P *<* *0.05). Although GHrCT2 maintained a proliferative trend through Days 2 and 3, it still revealed significant suppression compared to controls (*P *<* *0.01). By Day 3, GHrC sustained high proliferative enhancement, while GHrCT1 displayed a substantial increase in promotion (*P *<* *0.001). These results indicated the excellent cytocompatibility of hydrogel groups but also suggested that high-concentration TA incorporation might induce cytotoxic effects.

To further investigate the relationship between TA content and cytotoxicity, GHrCT4 and GHrCT8 groups with higher TA concentrations were introduced in addition to the previously established groups. The viable cell count, specifically labeled by FDA staining with bright green fluorescence, was quantified on Days 1, 3 and 5. As shown in Figure 3D, both the GHrCT4 and GHrCT8 groups exhibited significant cytotoxicity, as evidenced by a gradual reduction in the number of live cells, with almost no viable cells detectable by Day 5. In contrast, all other groups demonstrated clear cell proliferation. The results of FDA/PI staining were consistent with those obtained from the CCK-8 assay, confirming that the cytotoxicity of TA was concentration-dependent. Furthermore, hydrogels with lower TA concentrations maintained excellent biocompatibility.

To assess the *in vitro* cell migration capacity of the hydrogels, a scratch wound healing assay was conducted using L929 fibroblast cells. As illustrated in Figure 3E, significant differences in cell migration patterns were observed after 24 h of incubation. The GH, GHrC and GHrCT1 groups demonstrated enhanced cell migration activity, while the hydrogel formulation containing elevated concentrations of TA exhibited inhibitory effects on cell migration. The experimental results demonstrated strong consistency with the preceding findings obtained from both the CCK-8 assay and live/dead cell viability staining.

### *In vitro* antioxidant properties of hydrogels

The overproduction of ROS at wound sites induces significant oxidative stress, resulting in structural damage to essential cellular components, including proteins, lipids and DNA. This oxidative damage not only initiates inflammatory responses but also adversely affects critical wound healing processes [59]. Specifically, it impairs the functionality of endogenous stem cells and macrophages, suppresses angiogenesis and causes endothelial dysfunction. These cumulative effects exacerbate tissue damage and substantially hinder the wound regeneration process [60].

**Figure 4.**
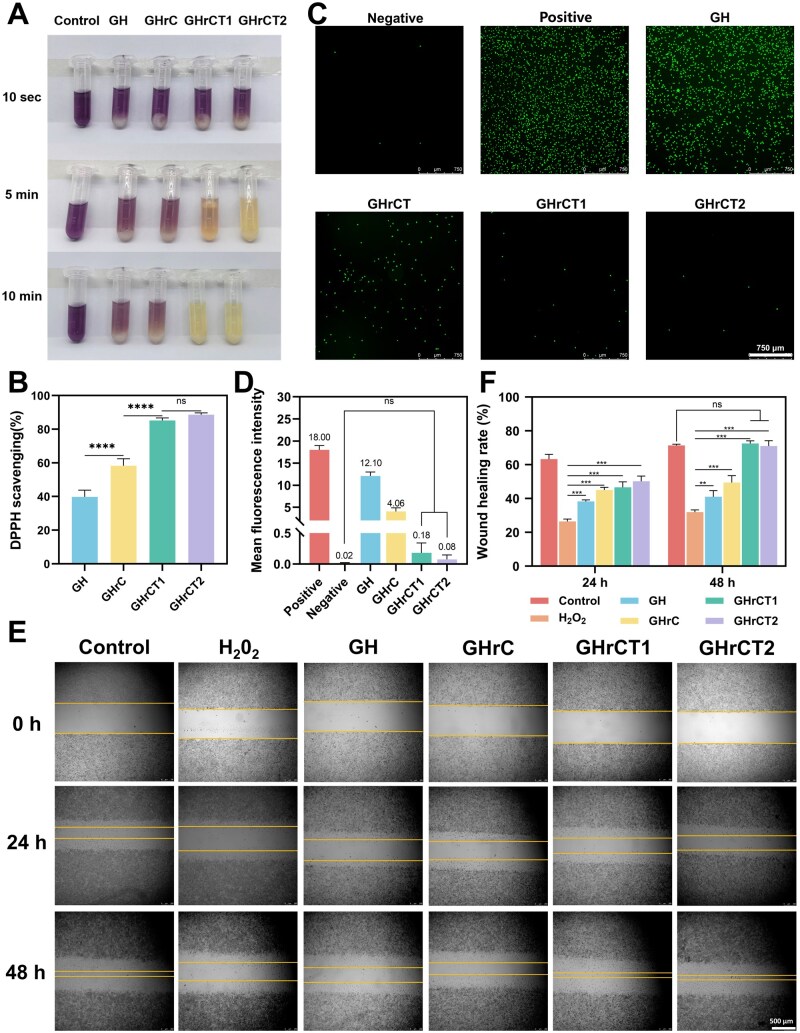
Antioxidant properties of hydrogels. (**A**) Hydrogel DPPH scavenging visualization. (**B**) Quantitative DPPH scavenging analysis. (**C**) Intracellular ROS imaging (DCFH-DA). (**D**) Cellular ROS scavenging efficiency. (**E**) Cell migration in oxidative stress environments: (E) photographs and (**F**) wound healing rates.

The GH and GHrC groups demonstrated moderate DPPH scavenging activity, whereas the GHrCT1 and GHrCT2 groups exhibited significantly enhanced antioxidant capacity, faster reaction kinetics and superior DPPH scavenging rates (Figure 4A and B). Notably, the GHrCT2 group achieved the maximum DPPH scavenging rate of 88.63%. Statistical analysis revealed no significant difference in DPPH scavenging activity between the GHrCT1 and GHrCT2 groups (*P* > 0.05), indicating that low TA loading is sufficient to achieve optimal antioxidant capacity.

Subsequently, intracellular ROS levels in hydrogel-cultured cells were quantitatively assessed using the DCFH-DA fluorescent probe, and antioxidant capacity evaluation was conducted through CLSM analysis. Quantitative fluorescence imaging revealed a significant reduction in staining intensity in the GHrC, GHrCT1 and GHrCT2 groups compared to both the positive control and GH groups (*P* < 0.05) (Figure 4C and D). Notably, the GHrCT1 and GHrCT2 groups exhibited the lowest fluorescence intensity, approaching levels comparable to the negative control group. These findings indicated the remarkable ROS-scavenging capability of GHrCT1 and GHrCT2 in L929 cells, which could be attributed to the controlled release of TA from the composite hydrogels. The experimental results collectively demonstrated that the GHrCT composite hydrogel system effectively mitigated intracellular oxidative stress and maintained cellular redox homeostasis.

Furthermore, the migratory capacity of injured L929 cells in response to hydrogel treatment was evaluated using a scratch assay under hydrogen peroxide (H_2_O_2_)-induced oxidative stress conditions, and the results were shown in Figure 4E. Quantitative analysis (Figure 4F) revealed that H_2_O_2_ stimulation significantly inhibited cell migration across all experimental groups compared to the control group (*P *<* *0.05). However, the GHrCT1- and GHrCT2-treated groups exhibited significantly enhanced cell migration rates compared to both H_2_O_2_-treated and GH groups on Days 1 and 2 (*P *<* *0.01), achieving levels comparable to the control group. The enhanced migratory capacity of damaged cells under oxidative stress conditions suggested the hydrogels’ effective ROS scavenging capability. These findings indicated that the GHrCT1 hydrogel accelerated wound healing through a dual mechanism of ROS scavenging and proliferation enhancement and demonstrated its potential as a novel therapeutic strategy for impaired wound repair.

### *In vivo* evaluation of hydrogel-mediated cutaneous wound healing

**Figure 5.**
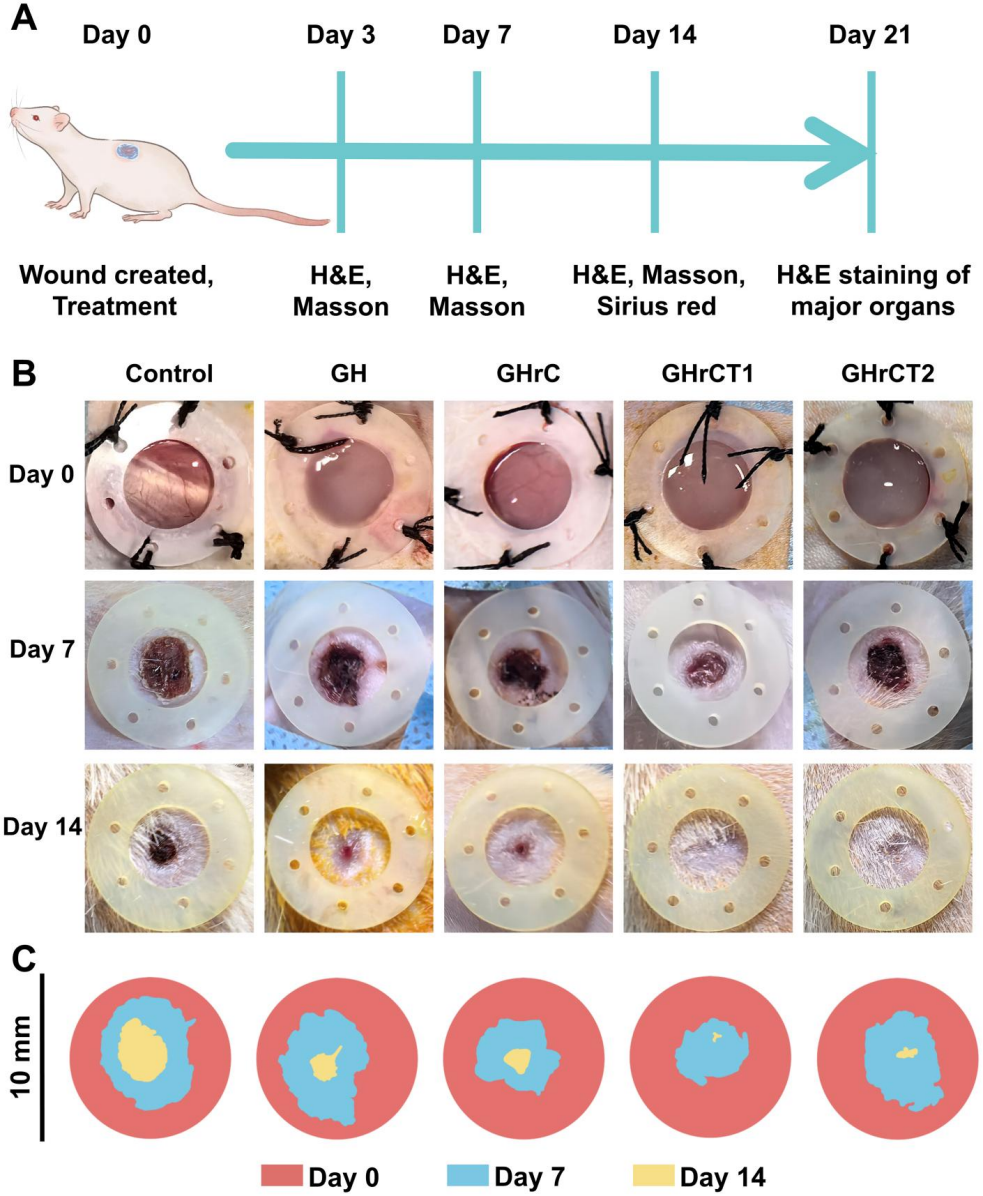
*In vivo* wound repair experiments with hydrogels. (**A**) Schematic timeline of the wound repair experiment in SD rats. (**B**) Representative macroscopic images of wound healing progression with different treatments. (**C**) Simulation plots of wound closure.

To comprehensively assess the therapeutic potential of the hydrogel in cutaneous wound healing, an *in vivo* study was conducted using SD rats. A standardized 10-mm full-thickness skin defect model was surgically created on the dorsal region of each animal. Figure 5A systematically illustrated the macroscopic wound healing progression across all experimental groups throughout the three-week observation period of skin regeneration.

During the initial 7-day postoperative period, wound sites in both the control and individual hydrogel treatment groups exhibited characteristic crust formation, and the GHrCT1 group demonstrated significantly reduced wound areas (Figure 5B). On Day 14 post-treatment, persistent skin lesions remained observable in the control, GH and GHrC groups, while the GHrCT1 and GHrCT2 groups showed substantial wound closure (Figure 5C). The *in situ* forming hydrogels demonstrated excellent conformability to wound contours, providing complete coverage and maintaining an optimal moist environment to facilitate skin wound healing. The superior wound healing performance observed in the GHrCT1 hydrogel group could be attributed to the synergistic effects of three key factors: (1) the inherent biocompatibility of the hydrogel matrix, (2) the regenerative potential of rhCol III and (3) the effective antioxidant activity of TA at low concentrations.

**Figure 6.**
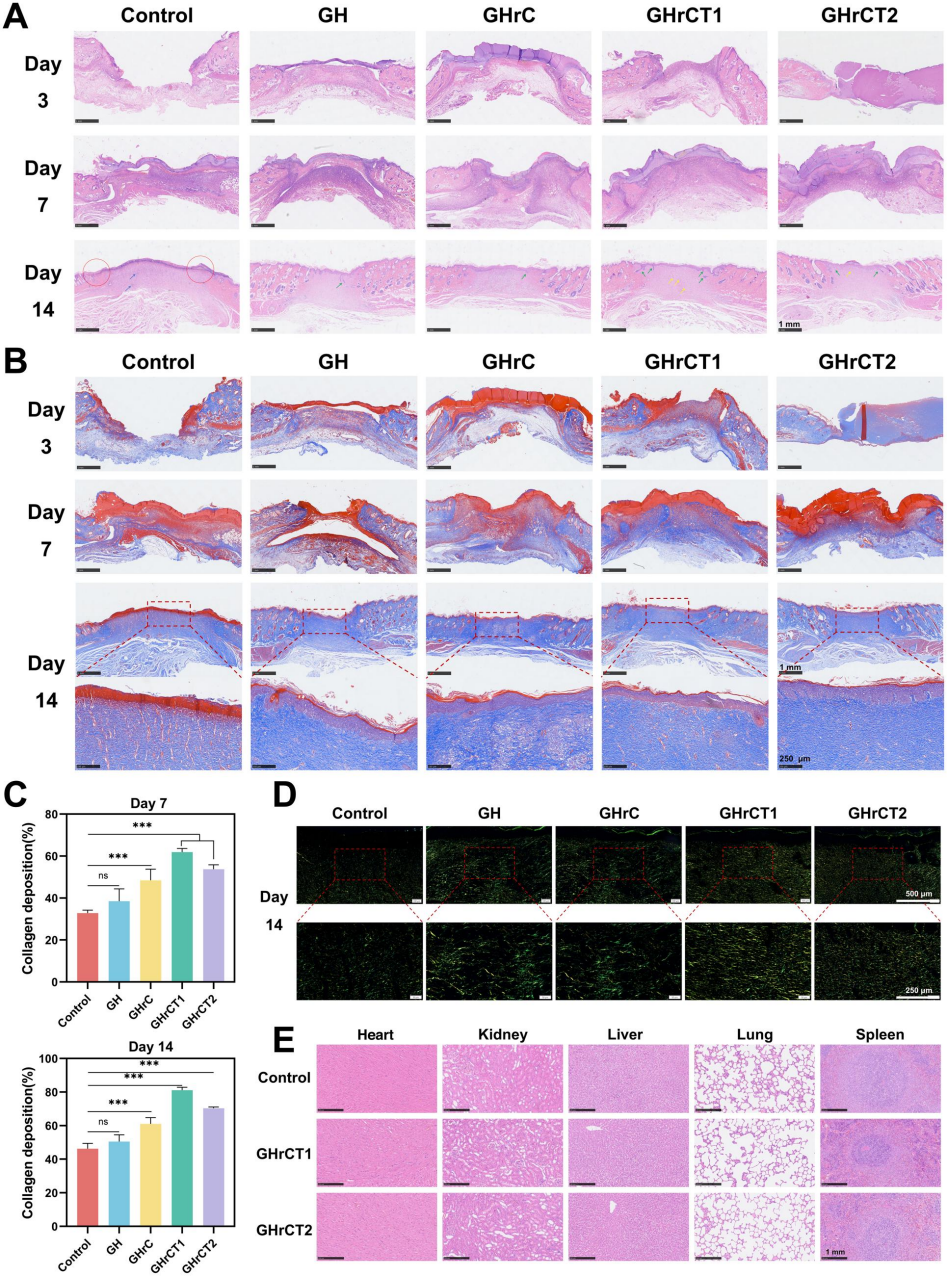
Histologic staining of hydrogels for *in vivo* wound repair experiments. (**A**) Histological evaluation by H&E staining. (**B**) Connective tissue architecture analysis via masson's trichrome staining. (**C**) Collagen deposition rate on postoperative Days 7 and 14. (**D**) Collagen fiber characterization using sirius red staining under polarized light. (**E**) H&E staining plots of major organs of rats after 21 days of different treatments.

As shown in Figure 6A, H&E staining revealed distinct healing patterns across experimental timelines. On Day 3 post-treatment, all groups exhibited pronounced tissue defects and substantial inflammatory cell infiltration, histopathological hallmarks of the acute inflammatory phase during early wound repair. By Day 7, while varying degrees of re-epithelialization were observed in all groups, persistent inflammatory cell infiltration remained prominent in the control, GH and GHrC groups, suggesting protracted chronic inflammatory responses. At Day 14, the control group still exhibited incomplete re-epithelialization (indicated by red circles) and persistent inflammatory cell presence (marked by blue arrows). In contrast, all other experimental groups had achieved complete re-epithelialization. Specifically, the GHrCT1 group demonstrated intact and uniform neo-epidermis with substantial neovascularization (yellow arrows) and formation of skin appendages such as hair follicles and sebaceous glands (green arrows).

The Masson staining results (Figure 6B and C) on Day 14 demonstrated that the control group exhibited abundant new keratinocytes with persistent re-epithelialization status while showing minimal collagen deposition. In contrast, both GHrCT1 and GHrCT2 groups displayed densely arranged collagen fibers with well-organized architecture comparable to normal skin tissue. Particularly, the GHrCT1 group presented a thin and uniform neo-epidermal layer accompanied by substantial neovascularization within the dermal layer, indicating active skin remodeling phase. According to the collagen deposition revealed by Masson staining on the 14th day, the collagen deposition rate in GHrC group was 31.84% higher than that in the control group, and the collagen deposition rate in GHrCT1 group was the highest, reaching 81.11%.

Sirius red staining (Figure 6D) exhibits specific binding affinity for collagen fibers, displaying distinct birefringence patterns under polarized light microscopy [61]. Type I collagen, appearing orange–yellow or red, represents the primary component of mature scar tissue. In contrast, type III collagen, exhibiting green birefringence, predominates in early granulation tissue. Analysis of Sirius red staining patterns revealed distinct collagen composition differences among the experimental groups. The control group had the least amount of newly generated collagen and most of it was predominantly type III collagen. Further, in the GH and GHrC groups, type III collagen with disorganized fibers was predominantly distributed, but more ordered type I collagen was also beginning to be produced. In contrast, both GHrCT1 and GHrCT2 groups exhibited progressive maturation characterized by densely packed type I collagen bundles demonstrating parallel orientation and enhanced birefringence patterns. These groups exhibited reduced type III collagen content accompanied by increased type I collagen deposition, showing progressively organized fiber arrangement.

Twenty-one days post-skin trauma surgery, H&E staining was performed on Control and GHrCT-treated rats with their major organs (heart, kidney, liver, lung and spleen) to assess the tissue safety of the multinetwork *in situ* forming hydrogel. By comparing the staining diagrams of the three groups of organs in Figure 6E, it was observed that all organs were structurally intact and clear, and no obvious lesions were seen.

*In vivo* experimental results demonstrated that the GHrCT hydrogel effectively formed *in situ* at wound sites, creating an optimal moist wound environment while providing essential mechanical support and protective barrier functions. Through synergistic interplay between TA and rhCol III, the hydrogel exhibited synergistic therapeutic effects, including: (1) efficient scavenging of excess ROS to mitigate oxidative stress-induced damage, (2) enhanced cellular migration and proliferation, (3) guided deposition of well-organized collagen matrices and (4) comprehensive promotion of the multistage wound healing cascade.

## Conclusion

This study established a covalent–noncovalent hybrid network system through the integration of GelMA-derived photo-crosslinked networks with intra-/intermolecular hydrogen-bond-dominated interactions among TA, rhCol III and HD. This innovative architectural design enabled multifunctional wound management by synergistically combining dynamic network reconfiguration capability for enhanced shape adaptability with energy-dissipative sacrificial bonding mechanisms that reinforce mechanical resilience. Building upon the native biocompatibility of Gel and HA, the GHrCT1 system demonstrated exceptional biocompatibility and tissue compatibility. The synergistic coordination between polyphenolic components and rhCol III endowed the GHrCT1 hydrogel with integrated antioxidative and pro-regenerative capacities, effectively neutralizing pathological oxidative stress while enhancing fibroblast motility and collagen deposition in ROS-rich wound microenvironments. *In vivo* experiments in an SD rat model of total skin defects showed that GHrCT1 hydrogels accelerated cell migration and enhanced angiogenesis to achieve accelerated wound healing. The focus of this study was on material design and functional validation, and although GHrCT hydrogels showed promising healing effects, the specific biomolecular mechanisms of wound healing needed to be further elucidated. Future work will combine transcriptomics and proteomics approaches to explore the molecular targets of GHrCT in mediating tissue regeneration. Furthermore, long-term comparative experiments for complex wounds will be performed against clinically established dressings to validate its clinical applicability. Overall, the GHrCT hydrogel demonstrates potential in accelerating the wound healing process.

## Data Availability

All data generated or analyzed during this study are included in this published article and its supplementary information files. Requests for materials related to this study should be directed to the corresponding author.
